# GAPDH Is a Novel Ferroptosis-Related Marker and Correlates with Immune Microenvironment in Lung Adenocarcinoma

**DOI:** 10.3390/metabo13020142

**Published:** 2023-01-17

**Authors:** Xiaohu Ouyang, Rui Zhu, Lan Lin, Xunxun Wang, Qigang Zhuang, Desheng Hu

**Affiliations:** 1 Department of Integrated Traditional Chinese and Western Medicine, Union Hospital, Tongji Medical College, Huazhong University of Science and Technology, Wuhan 430022, China; 2 The First Clinical College, Tongji Medical College, Huazhong University of Science and Technology, Wuhan 430022, China; 3 Key Laboratory of Biological Targeted Therapy, The Ministry of Education, Wuhan 430022, China; 4 Clinical Research Center of Cancer Immunotherapy, Union Hospital, Tongji Medical College, Huazhong University of Science and Technology, Wuhan 430022, China

**Keywords:** ferroptosis, lung adenocarcinoma, tumor immune microenvironment, immunotherapy, prognosis, GAPDH

## Abstract

Lung adenocarcinoma (LUAD) is a prevalent form of lung cancer with high morbidity and fatality rates. Ferroptosis is a type of programmed cell death that is iron-dependent. Recent findings have suggested that ferroptosis inducers have promising prospects for the therapy of LUAD. However, ferroptosis-related gene expression in LUAD and its relationship with the tumor prognosis and tumor immune microenvironment remain unknown. We identified a total of 638 ferroptosis-related genes, built a LUAD ferroptosis-related risk model (FRRM) with the help of Least Absolute Shrinkage Selection Operator (LASSO) regression analysis based on The Cancer Genome Atlas (TCGA) database, split LUAD patients into high- and low-risk clusters, and verified the model utilizing the Gene Expression Omnibus (GEO) database. The results of the FRRM’s principal component analysis (PCA) demonstrated its strong predictive power. Further, univariate and multivariate Cox and AUC curve analyses demonstrated that the model was independent of other clinical traits and served as an independent prognostic factor. The nomogram demonstrated strong predictive power for overall survival, according to calibration plots. We also explored variations in clinical characteristics, immune cell infiltration, immune-related function, and functional pathways between the high- and low-risk groups. Additionally, we used a protein–protein interaction (PPI) network of various genes in the two groups to search for potential target genes. GAPDH was then chosen for a follow-up investigation. An analysis was performed on the relationship between GAPDH and variations in survival prognosis, clinical traits, immune cell infiltration, immune checkpoints, and immunotherapy. In vitro tests further supported the probable functions of GAPDH as a ferroptosis marker in LUAD. In conclusion, a novel ferroptosis-related prognostic gene, GAPDH, was discovered, whose expression was connected to the tumor immune microenvironment. The combination of immunotherapy and the targeting of GAPDH to induce ferroptosis in LUAD may provide a novel therapeutical option.

## 1. Introduction

Lung cancer is the most prevalent and potentially lethal malignancy throughout the world, as well as the major driver of cancer-related mortality, with an annual estimation of millions of new cases and fatalities, accounting for roughly one-quarter of all cancer deaths [1,2]. According to estimates, between 80% and 85% of all lung malignancies are non-small cell lung cancers (NSCLCs), with the three main subtypes being adenocarcinoma, squamous cell carcinoma, and large cell carcinoma [1]. Squamous cell carcinoma was the most prevalent pulmonary cancer histologic subtype prior to the 1990s, especially among men. Since then, the incidence of lung adenocarcinoma (LUAD) has outpaced that of squamous cell carcinoma and emerged as the predominant histologic subtype [3]. The incidence and mortality of LUAD are rising every year, which has an average 5-year survival rate of only 15% of patients [4]. It is still a severe clinical issue because LUAD patients’ prognoses drastically differ from one another [5]. The targets connected to the occurrence and progression of LUAD have so far eluded researchers. Finding promising biomarkers, reliable therapeutic targets, and new prognostic indicators for LUAD is crucial.

Reactive oxygen species (ROS) and cytotoxic lipid buildup culminate in the iron-dependent form of programmed cell death known as ferroptosis, which causes lipid peroxidation of the cellular membrane [6]. A wide range of biological processes, including development, aging, immunology, and cancer, have been linked to ferroptosis [7]. Additionally, it is thought that ferroptosis resistance aids in the development of tumors [6]. On the contrary, the activation of ferroptosis increases the effectiveness of cancer therapies such as immune checkpoint blockade, radiation, and chemotherapy [8]. Ferroptosis is controlled by a variety of cellular metabolic pathways, including amino acid metabolism, redox homeostasis, and mitochondrial activity [9]. In parallel to the metabolism of lipids and amino acids, the effect of glucose, a key fuel source for energy metabolism, also needs to be investigated in the ferroptosis process of cancer cells [10].

There are ten different enzymes involved in the conversion of glucose to pyruvate during aerobic glycolysis, but glyceraldehyde-3-phosphate dehydrogenase (GAPDH) is of particular relevance. GAPDH, an enzyme for glycolysis, simultaneously activates the oxidation and phosphorylation of glyceraldehyde-3-phosphate to produce 1,3-biphosphoglycerate and NAD+ as an electron acceptor. As a typical moonlighting protein, GAPDH serves a plethora of roles in cells and takes part in a number of crucial chemical cascades. GAPDH participates in the cell’s response to numerous cytotoxic or harmful conditions, including oxidative stress [11], starvation [12], proteotoxic stress [13], and the toxicity of chemical agents [13], in addition to carrying out the functions required for normal cell physiology. Recent research has shown that GAPDH may serve as a rate-limiting mechanism in the setting of Warburg effects [14]. Although GAPDH is overexpressed in several malignancies, it is still unknown how it contributes to carcinogenesis [15,16]. A few reports indicate that proliferation and invasion are linked to increased GAPDH expression in some tumor cells [16,17,18]. Additionally, it was discovered that chronic myeloid leukemia (CML) cells overexpressed GAPDH, contributing to CML resistance to chemotherapy [19]. It is noteworthy that the overexpression of GAPDH, but not of the other glycolytic enzymes studied, promoted tumor cell survival and resistance to chemotherapy in caspase-independent cell death by preserving a small number of intact mitochondria [20]. Reduced GAPDH expression in tumors slows the glycolytic pathway, interfering with the energy metabolism of the tumor and enhancing the death of tumor cells caused by ferroptosis [21]. However, further research is still needed to investigate the direct relationship between GAPDH and ferroptosis.

Herein, we used TCGA datasets to analyze the expression pattern of ferroptosis-associated genes and then built a risk prediction model. Through variance analysis, GAPDH was found to be associated with ferroptosis in LUAD patients. Furthermore, we demonstrated that GAPDH, which is highly expressed in LUAD patients and associated with the tumor immune microenvironment (TIME), may serve as a new diagnostic and therapeutic target. GAPDH was selected for further functional validation in H1299 and A549 cells in vitro. Overall, our research offers a fresh understanding of the connection between GAPDH and ferroptosis.

## 2. Materials and Methods

### 2.1. Data Acquisition and Patient Characteristics

The raw RNA sequencing (RNA-seq) data profiles of 551 LUAD samples were retrieved using the TCGA database (https://portal.gdc.cancer.gov/, accessed on 1 May 2022). In addition, the TCGA database included clinical information on 486 LUAD samples, such as age, gender, grade, and prognostic variables. The original microarray data of 442 LUAD samples containing clinical information from the GSE68465 cohort were downloaded from the Gene Expression Omnibus (GEO) database (https://www.ncbi.nlm.nih.gov/geo/, accessed on 20 May 2022), and we applied strawberry perl to standardize them. Subsequently, 580 LUAD mutated samples were obtained from the TCGA database. Images of immunohistochemistry were obtained from the human protein atlas (HPA) database (https://www.proteinatlas.org/, accessed on 16 September 2022). A total of 574 LUAD clinical patients with immune scoring were downloaded from The Cancer Immunome Atlas (TCIA) database.

### 2.2. Analysis of Differential Expression of Genes Related to Ferroptosis

A total of 582 and 67 ferroptosis-related genes were obtained from the GeneCards and OMIM databases, respectively. According to the screening criteria, differentially expressed genes (DEGs) in LUAD were discovered using the R “Limma” package (*p* < 0.05).

### 2.3. Construction and Validation of a Ferroptosis-Related Risk Model (FRRM)

Using univariate Cox regression techniques on the TCGA cohort, the genes linked to prognosis were chosen from the DEGs linked to ferroptosis, and the cutoff point was set to a *p*-value < 0.05. The “maftools” R package was applied to evaluate the relationship between the frequencies of gene mutations in the training set samples and the mutated samples. Least Absolute Shrinkage Selection Operator (LASSO) Cox regression analysis was used to establish a prognostic risk score model for predicting overall survival in LUAD samples based on the TCGA database. The following was the risk score formula [22]:

$$\mathrm{risk}\;\mathrm{score}=\sum_{1}^{i}\mathrm{Coefi}*\mathrm{ExpGenei}$$

The “glmnet” and “survival” R packages were applied for genes connected to the prognosis of ferroptosis in LUAD. “Coef” stands for non-zero regression coefficients derived from the LASSO Cox regression analysis, and the expression values of the genes derived from the prognostic risk score model are represented by “ExpGene”. LUAD patients were split into high- and low-risk categories depending on the median ferroptosis risk score of the TCGA-LUAD cohort sample, and the “limma” package was used for principal component analysis (PCA) of gene expression to identify remarkable variations between the two categories in the TGGA and GEO databases. The K-M approach was also utilized to determine whether the survival prognosis varied between the high- and low-risk groups. Utilizing the GEO database, the model’s viability was further confirmed by univariate and multivariate Cox and ROC analyses. Kaplan–Meier curves and nomograms were plotted to evaluate the discriminative capacity of clinicopathological features.

### 2.4. Immune-Related Characteristics and GSVA of FRRM

The “limma”, “ggpubr”, and reshape2” packages were used to estimate the association between the risk score and immune cell infiltration as well as that between the risk score and immune-related function. Using the “GSVA” and “GSEABase” packages, we further investigated whether the high- and low-risk groups had different pathways or functions.

### 2.5. Identification of the DEGs between the High- and Low-Risk-Score Groups

Relying on the screening criteria, the “Limma” package in R was used to find 409 DEGs in LUAD (*p* < 0.05). We used Cytoscape to visualize the interaction network data that we created using the STRING database to generate a protein–protein interaction network (PPI) pertaining to DEGs. Using the Cytoscape cubHubba plugin, the top 10 most pooled hub genes were examined (version: 3.9.1).

### 2.6. The Related Characteristics of GAPDH

The TIMER2.0 (http://timer.cistrome.org/, accessed on 9 June 2022) online database was applied to analyze the differential expression of GAPDH in pan-cancer. The “limma” and “ggpubr” R packages were applied to investigate the connection between target gene expression and the clinical characteristics of LUAD patients. The packages “CIBERSORT. R” and “limma” were applied to explore the penetration of 22 lymphocyte-associated target cells in the tumor microenvironment. Subsequently, the relationships between GAPDH expression and the tumor immune microenvironment, immune cell infiltration, immune checkpoints, and the tumor mutation burden were explored using the “corrplot”, “limma”, “reshape2” and “ggpubr” packages.

### 2.7. Immunotherapy

The intersection samples of TCIA (574 samples) and TCGA (551 samples) were applied for immunotherapy analysis. The R language loaded with the packages “limma” and “ggpubr” was used for immunotherapy analysis in low and high GAPDH expression groups.

### 2.8. Cell Lines and Reagents

HBE, LLC, A549, and H1299 cells were obtained from the Department of Respiratory and Critical Care Medicine, Wuhan Union Hospital, Tongji Medical College, Huazhong University of Science and Technology. All cells were grown in complete medium (supplemented with 10% fetal bovine serum (FBS; Life Technologies, Carlsbad, CA, USA) and Penicillin–Streptomycin (100 U/mL)) at 37 °C with 5% CO_2_ in a humidified incubator. HBE and LLC cells were cultured in DMEM (Gibco, Waltham, MA, USA) complete medium. H1299 and A549 cells were grown in RPMI 1640 (Gibco, Waltham, MA, USA) complete medium. Erastin, RSL3, and ferrostatin-1 were purchased from Selleck Chemicals. The following treatments were applied to several cell groups: the control group (no processing), the erastin treatment group (5 μM or 10 μM), the RSL3 treatment group (2.5 μM or 5 μM), and the ferrostatin-1 treatment group (10 μM).

### 2.9. Light Microscopy and Immunofluorescence Microscopy

Cells were cultured in 6-well plates and given the recommended dose of erastin (5 μM or 10 μM) for light microscopy. A Leica microscope with a 10× phase-contrast objective was used to take the phase-contrast pictures. Cells were sown in 6-well plates and grown for 24 h prior to immunofluorescence microscopy. Additionally, after draining the medium with PBS, cells were permeabilized with 0.5% Triton X-100 in PBS for 15 min after being fixed with 4% paraformaldehyde for 15 min, and then washed with PBS three times for five minutes each. Then, 3% BSA in PBS was used to further block cells for 30 min. The cells were then rinsed three times with PBS for five minutes each after being incubated with the primary antibody anti-HMOX1 (1:100) (catalog number A1346; Abclonal, Wuhan, Chian; dilution, 1:1000) all night long at 4 °C. The cells were then treated with fluorescently tagged secondary antibodies (catalog number AS007; Abclonal, Wuhan, China; dilution, 1:100) for 45 min at room temperature before being washed away three times with PBS for five minutes each, and the nuclei were then marked with DAPI. Finally, a fluorescence microscope from Olympus was used to find fluorescently labeled target proteins.

### 2.10. RNA Isolation and Real-Time PCR

Total RNA from erastin-treated A549 and H1299 cells was extracted using TRIzol reagent (Life Technologies), and according to the manufacturer’s instructions, Primescript first-strand cDNA synthesis kit (Takara, Dalian, China) was used to turn the extracted RNA into cDNA. The SYBR-Green kit (Takara, Dalian, China) was applied to undertake real-time PCR in order to measure the levels of ferroptosis genes’ mRNA expression. The computation is based on the 2-∆∆CT approach, and β-actin was used as a comparison to normalize the expression and evaluate the relative expression of each group. The real-time PCR primers were purchased from Tsingke Biotechnology and are provided in Table S1.

### 2.11. Western Blotting

The cell lysates were prepared in lysis buffer (Beyotime Biotechnology, Shanghai, China), and the BCA kit (Beyotime Biotechnology, Shanghai, Chian) was utilized to calculate the protein concentration. The samples were loaded onto a sodium dodecyl sulfate (SDS)-PAGE gel and thereafter electrotransferred to a PVDF membrane (Millipore, Bedford, MA, USA). The membrane was blocked with 5% nonfat milk for 1 h, then incubated with primary antibodies for 16 h at 4 degrees Celsius, and washed three times with tris-buffered saline with Tween (TBST) for 10 min each, after which the membrane was incubated with the HRP-conjugated goat anti-rabbit IgG antibody (cat no. AS014; Abclonal, Wuhan, Chian; dilution, 1:4000) for 1 h at room temperature. The chemical reaction was visualized using Clarity Western ECL substrate (Bio-Rad, Hercules, CA, USA) and identified by exposure to an autoradiographic film. The antibodies applied included those against ACTIN (cat no. AC006; Abclonal, Wuhan, Chian; dilution, 1:1000), GAPDH (cat no. AC001; Abclonal, Wuhan, Chian; dilution, 1:1000), HMOX1 (cat no. A1346; Abclonal, Wuhan, Chian; dilution, 1:1000), SLC7A11 (cat no. A13685; Abclonal, Wuhan, Chian; dilution, 1:1000), and GPX4 (cat no. A1933; Abclonal, Wuhan, Chian; dilution, 1:1000). 

### 2.12. Statistical Analysis

R version 4.1.2 or Graphpad Prism 8 was used to analyze all data after each experiment was run at least three times. Results are expressed as means ± standard error of the mean (SEM). One-way variance (ANOVA) was employed to assess group differences. Kaplan–Meier curves and the log-rank test were used to analyze the differences in survival rates among various risk groups. At *p* < 0.05, differences were declared statistically significant.

## 3. Results

This investigation included 551 LUAD patients from the TCGA database and 442 LUAD patients from the GEO database. Figure S1 presents the general design and process flow of the project.

### 3.1. Establishment of the Prognostic Model on the Training Set

**Figure 1:**
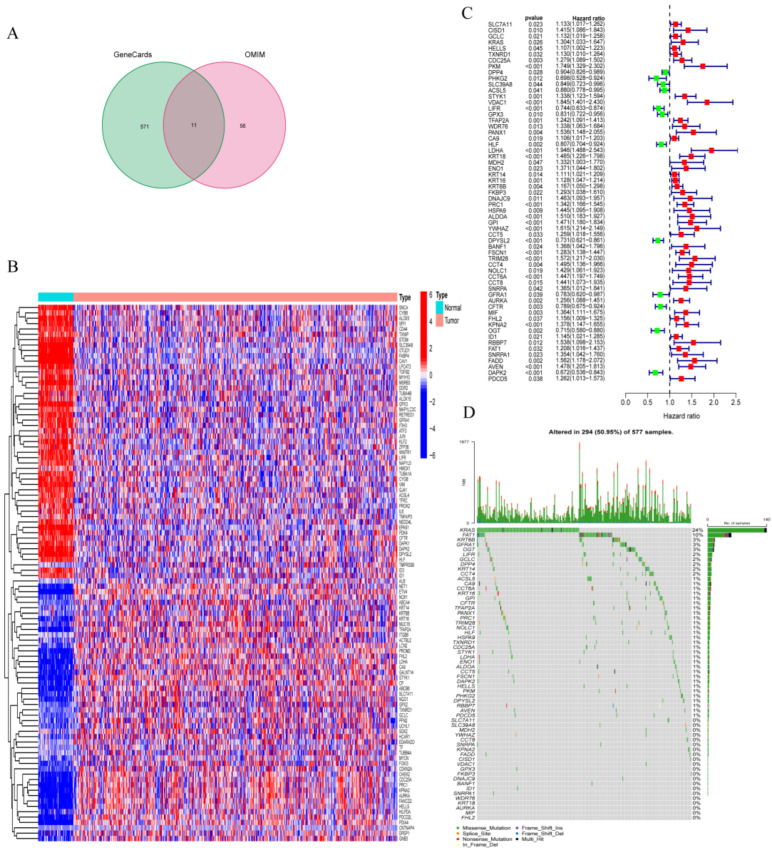
Genes connected to ferroptosis in LUAD. (**A**) Venn diagram displaying the 638 ferroptosis-related genes obtained from GeneCards and OMIM databases. (**B**) Heatmap displaying the top 50 DEGs’ expression levels in LUAD and normal lung tissues. DEGs, differentially expressed genes. (**C**) Ferroptosis–related genes studied in univariate analysis. If a gene’s hazard ratio is greater than 1, it means that the gene acts as a risk factor for the corresponding tumor, and vice versa. (**D**) The frequency of mutations in 60 genes in LUAD patients from the TCGA cohort.

GeneCards and OMIM databases contained a total of 638 ferroptosis-related genes (FRGs), among which 11 genes were regulated genes common to both databases, 571 genes were only in GeneCards, and 56 genes were only in OMIM database (Figure 1A, Table S2). Then, 248 abnormally expressed genes were screened out from TCGA datasets after examining the transcriptional activity of FRGs in tumor and normal samples (FDR 0.05, logFC = 0.585). It comprised 61 upregulated genes and 187 downregulated genes (Figure 1B).

As a training set, the TCGA-LUAD cohort was applied. The 248 FRGs were screened from the TCGA-LUAD cohort. Sixty FRGs associated with patient survival were identified using univariate Cox analysis. Among them, DAPK2, LIFR, and DPYSL2 were connected to a good prognosis for patients (*p* < 0.001), whereas the high expression of YWHAZ, ALDOA, KPNA2, GP1, TRIM28, AVEN, CCT6A, PKM, KRT18, FSCN1, PRC1, VDAC1, and LDHA (*p* < 0.001) was associated with a poor prognosis for patients (Figure 1C). The somatic mutation profiles of 60 genes involved in ferroptosis were then summarized. As shown in Figure 1D, the somatic mutation profiles of 39 prognosis-related genes showed a mutation frequency of 50.95% in the 577 LUAD samples, with KRAS and FAT1 having the highest mutation frequencies, while the remaining 21 were not mutated. Further research revealed a mutational co-occurrence association between VDAC1 and MDH2 and YWHAZ; SLC7A11 and NOLC1; CDC25A and FAT1 and DPP4; ACSL5 and FAT1 (Figure S2A). Subsequent establishments were made using the LASSO logistic regression technique based on the TCGA-LUAD cohort using these 60 genes. A total of 12 genes were obtained to build the ferroptosis-related risk model (FRRM) (Figure S2B,C), namely, VDAC1, LIFR, TFAP2A, LDHA, KRT18, PRC1, DPYSL2, FSCN1, TRIM28, OGT, ID1, and AVEN. Then, the patients were split into high- and low-risk groups based on median risk scores, and PCA analysis of the TCGA-LUAD and GEO-LUAD cohorts showed that these two groups could be clearly discriminated (Figure S2D,E).

### 3.2. Validation of the Prognostic Model with Clinical Features in LUAD Patients

**Figure 2:**
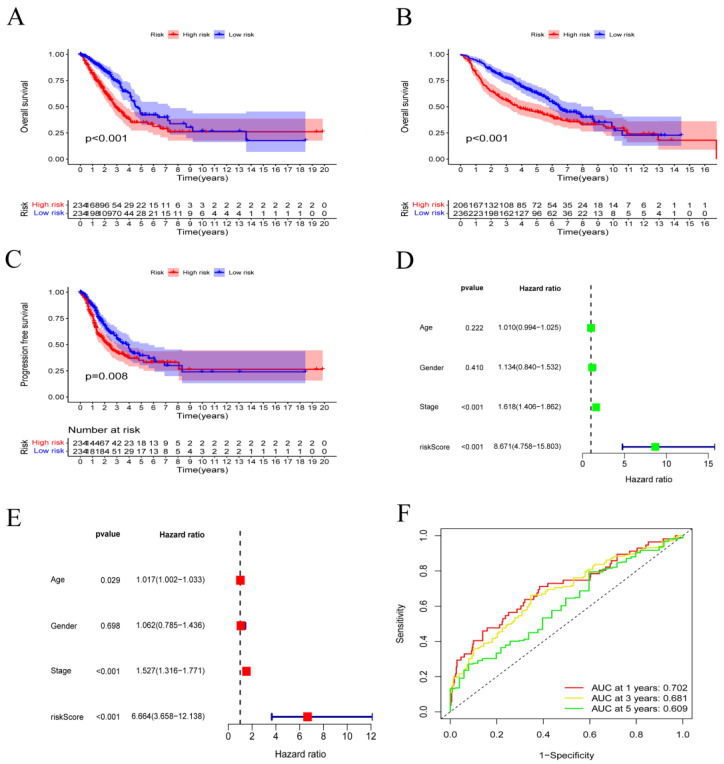
Survival analysis of ferroptosis−related risk score. (**A**) Overall survival in the TCGA–LUAD cohort according to the ferroptosis−related risk score. (**B**) Overall survival by ferroptosis-related risk score in the GEO-LUAD cohort. (**C**) PFS by ferroptosis-related risk score in the TCGA-LUAD cohort. (**D**) Results of the TCGA–LUAD cohort’s univariate Cox analysis. (**E**) Results of the TCGA–LUAD cohort’s multivariate Cox analysis are shown. (**F**) The TCGA−LUAD cohort’s AUC values at 1, 3, and 5 years.

**Figure 3:**
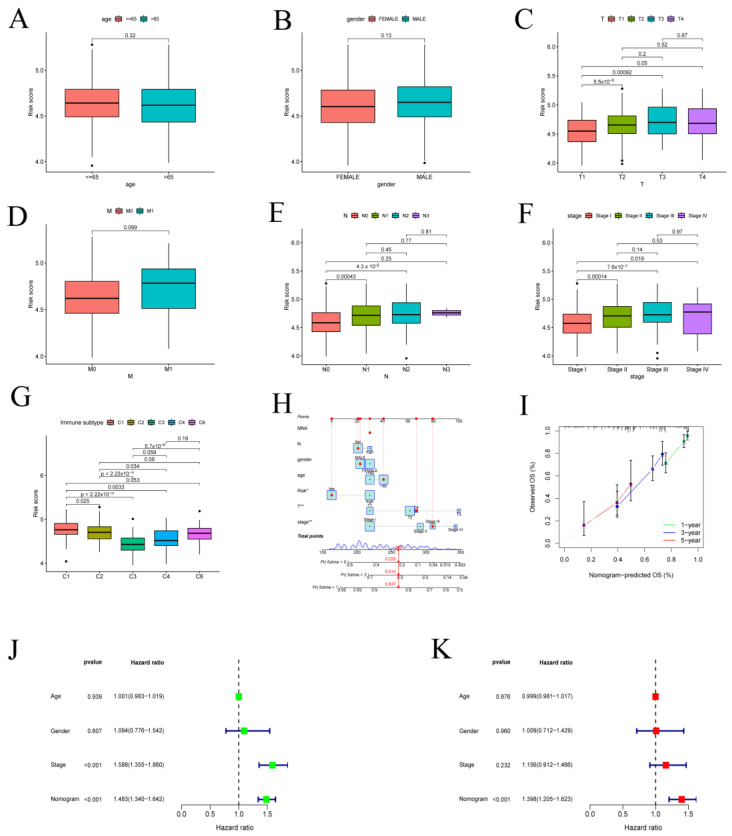
Ferroptosis−related risk score and clinical characteristics. (**A**–**F**) The relationship between ferroptosis-related risk score and clinicopathological traits, including TNM stage (Stage), tumor invasion (T), lymphoid metastasis (N), distal metastasis (M), gender (Gender), and age (Age), respectively, by Kruskal–Wallis rank sum test. (**G**) The association of ferroptosis-related risk score and immune subtypes by Kruskal–Wallis rank sum test. (**H**) Nomogram for the TCGA–LUAD cohort to forecast 1-, 3-, and 5-year OS. (**I**) In the TCGA–LUAD cohort, the calibration plot is used to evaluate how well the nomogram predicts outcomes for one, three, and five years. (**J**) Univariate Cox analysis. (**K**) Multivariate Cox analysis.

The prediction performance of the whole TCGA and GEO set was assessed for the purpose of confirming the robustness of this risk model. On the basis of the cutoff value, patients in these cohorts were divided into high- and low-risk categories. Depending on K-M curves, the overall survival (OS) of high-risk people in these cohorts was less than that of the low-risk group in both databases (*p* < 0.05) (Figure 2A,B). Similar to this, PFS (progression-free survival) was shorter in the high-risk group compared to the low-risk people (Figure 2C). The LUAD set underwent additional univariate and multivariate Cox analyses, which identified that the FRRM was an independent prognostic predictor (*p*-value < 0.001) (Figure 2D,E). To further assess the applicability of the FRRM, an AUC curve analysis was performed. The AUC for predicting OS was 0.702 at 1 year, 0.681 at 3 years, and 0.609 at 5 years (Figure 2F). We also analyzed how the various clinicopathological parameters related to the risk ratings. Among some individual characteristics, we discovered no statistically significant variations in the risk score according to age, gender, and distal metastasis (M). Patients with advanced tumor invasion (T), lymphoid metastasis (N), and TNM stage (Stage), however, had noticeably higher risk scores (Figure 3A–F). An immune subtype analysis revealed that subtype C3 patients had the lowest risk scores, whereas subtype C1 patients had the highest risk scores (Figure 3G). Stage, age, risk score, and gender were prognostic parameters that were incorporated into the nomogram to quantify the prognostic impact of the nomogram on 1-, 3-, and 5-year OS in LUAD patients (Figure 3H). Furthermore, the calibration charts nearly followed the ideal curve (Figure 3I). An independent prognostic analysis demonstrates that the nomogram may serve as an independent prognostic factor independent of other clinical features under both univariate and multivariate conditions (Figure 3J,K).

### 3.3. Immune System Characteristics and PPI Network in the High- and Low-Risk Groups

**Figure 4:**
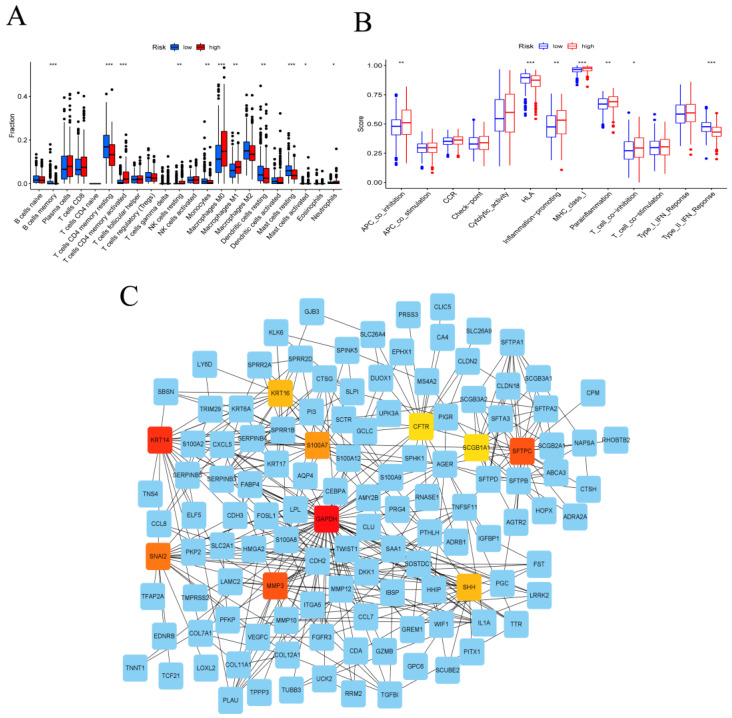
Protein−protein interaction (PPI) graph and correlation analysis between the tumor microenvironment characteristics and the risk score. (**A**) Immune infiltration of specific immune cell types in the TCGA−LUAD cohort patients with high and low risk. (**B**) Analysis of immunological function in the TCGA−LUAD cohort’s high-risk and low−risk patients. (**C**) Identification of the core genes with the highest connectivity in the subnetwork (* *p* < 0.05, ** *p* < 0.01, *** *p* < 0.001).

Immune cell variation analysis revealed immunological variations between the high- and low-risk groups, with considerably higher levels of activated memory CD4 T cells, resting NK cells, M0 macrophages, M1 macrophages, activated mast cells, and neutrophils in the high-risk group and significantly increased memory B cells, memory CD4 T cells, monocytes, resting dendritic cells, and resting mast cells in the low-risk group (Figure 4A). The analysis of immune-related function showed that APC coinhibition, inflammation promotion, MHC class I, parainflammation, and T-cell coinhibition were active in the high-risk group, while HLA and Type II IFN responses were active in the low-risk group (Figure 4B). Following that, GSVA analysis was adopted to investigate the differential expression of pathways in high- and low-risk groups. Most glycometabolism pathways, including pentose glycolysis, galactose, fructose, and mannose metabolism pathways, cysteine and methionine metabolism pathways, and the p53 signaling pathway, were discovered to be enriched in the high-risk score. In terms of the low-risk score, lipid metabolism-related pathways such as primary bile acid biosynthesis, fatty acid metabolism, alpha-linolenic acid metabolism, arachidonic acid metabolism, and linoleic acid metabolism were activated (Figure S3A).

We further identified 409 DEGs in the high- and low-risk score groups (FDR < 0.05, logFC > 1), with 177 DEGs being highly enriched in the high-risk group and 232 DEGs being highly enriched in the low-risk group. Figure S3B depicts the differential gene PPI network. We identified the genes with the most connections as key genes in the subnetwork using the Cytoscape plugin cytoHubba (Figure 4C). We chose ten genes from the network and sorted them by the order of connected nodes: GAPDH, KRT14, MMP3, SFTPC, SNAI2, S100A7, KRT16, SHH, CFTR, and SCGB1A1 (Figure 4C). GAPDH was chosen for further analysis because, as shown by GSVA analysis in Figure 4C, risk genes were primarily enriched in pathways related to glycometabolism.

### 3.4. GAPDH with Clinical Characteristics in LUAD

**Figure 5:**
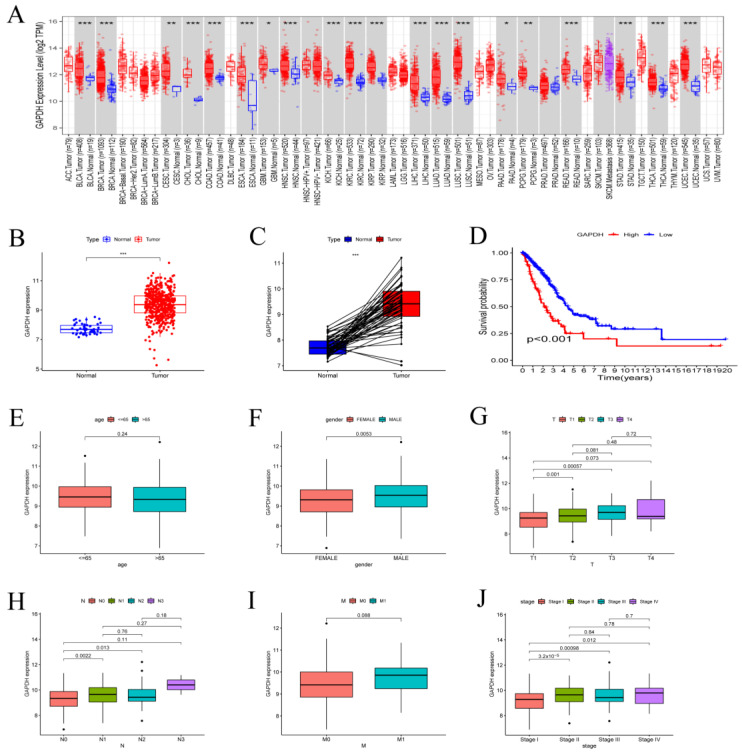
Differential expression of GAPDH and relationship between GAPDH and clinical characteristics. (**A**) Differential expression of GAPDH in pan−cancer. (**B**) Differential expression of GAPDH in LUAD by Wilcoxon rank sum test. (**C**) GAPDH expression was compared in normal and tumor samples derived from the same patient using paired differentiation analysis by Wilcoxon rank sum test. (**D**) Survival analysis of GAPDH expression in the TCGA−LUAD cohort. (**E**–**J**) The relationship between GAPDH and clinicopathological features, including TNM stage (Stage), tumor invasion (T), lymphoid metastasis (N), distal metastasis (M), gender (Gender), and age (Age), respectively, by Kruskal−Wallis rank sum test (* *p* < 0.05, ** *p* < 0.01, *** *p* < 0.001).

The TIMER2.0 database (http://timer.cistrome.org, accessed on 4 August 2022) was applied for correlation analysis between GAPDH and pan-cancer (Figure 5A). GAPDH was significantly different in 23 tumors, with all tumors showing significantly higher levels of GAPDH compared to normal tissues. Regarding LUAD, the Wilcoxon rank sum test showed that GAPDH expression was considerably higher in tumor samples than it was in normal samples (Figure 5B), and the pairing analysis between the normal and tumor tissues from the same patient produced similar results (Figure 5C). The survival analysis also revealed that high GAPDH expression in LUAD patients resulted in a lower survival time than low GAPDH expression (Figure 5D). We discovered no statistically significant changes in GAPDH expression between various ages and the M classifications of TNM stages (Figure 5E,I). However, compared to female patients, male patients exhibited considerably higher GAPDH expression (Figure 5F). We noticed that GAPDH expression rose progressively as the ranks moved up in the T and N categorization of the TNM stages (Figure 5G,H). The same condition also appears in different stages (Figure 5J). These findings unmistakably demonstrated that GAPDH expression in LUAD patients was inversely linked to their prognosis.

### 3.5. GAPDH Associated with Immune Cell Infiltration and Tumor Immunotherapy

**Figure 6:**
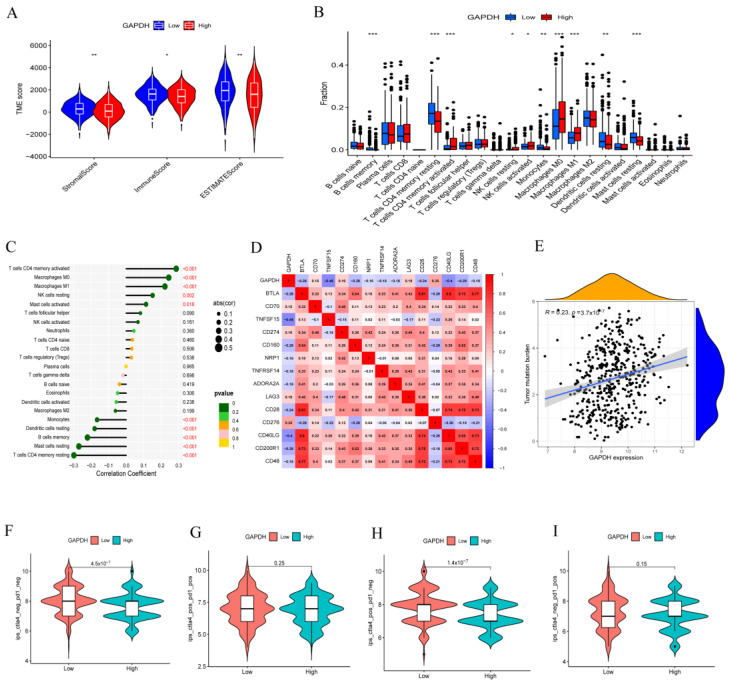
Tumor immune microenvironment and immunotherapy. (**A**) TME score between two groups by Wilcoxon rank sum test. (**B**) Immune cell variation analysis in high and low GAPDH expression groups. The significance test was conducted using the Wilcoxon rank sum test. (**C**) Immune cell correlation analysis in high and low GAPDH expression groups. (**D**) Correlation analysis between GAPDH and immune checkpoint genes. (**E**) Correlation analysis between GAPDH and overall tumor mutations. (**F**–**I**) Immunotherapy analysis between two groups (po, positive; neg, negative) by Wilcoxon test (* *p* < 0.05, ** *p* < 0.01, *** *p* < 0.001).

In the subsequent experiment, we deepen our research into the immunological and other TIME characteristics in LUAD patients with high or low GAPDH expression to identify potential treatment targets. Differential analysis of the tumor microenvironment showed that the high GAPDH expression group had lower levels of immune cells and stromal cells (Figure 6A). Immune cell infiltration displayed immunological differences between the high and low GAPDH expression groups, with significantly elevated numbers of activated memory CD4 T cells, resting NK cells, activated NK cells, M0 macrophages, and M1 macrophages in the high GAPDH expression group, and memory B cells, resting memory CD4 T cells, monocytes, resting dendritic cells, and resting mast cells were increased in the low GAPDH expression group (Figure 6B,C). Unexpectedly, the immune cell infiltration predicted by the prognostic model discussed above was similar to this result, corroborating the validity of both findings. GAPDH expression was found to be negatively correlated with the majority of immune checkpoint proteins, such as BTLA, TNFSF15, CD160, NRP1, TNFRSF14, ADORA2A, CD28, CD40LG, CD200R1, and CD48, and only a small number were found to be positively correlated with high GAPDH expression, including CD70, CD274, LAG3, and CD276 (Figure 6D). This demonstrates that increased GAPDH expression has the potential to suppress immunological checkpoint functionality. Subsequently, we discovered a positive correlation between high GAPDH expression and the number of overall tumor mutations (Figure 6E). In summary, increased GAPDH expression predicts a suppressive tumor immune microenvironment. PD-1/PD-L1 axis-targeting monoclonal antibodies are currently being successfully employed in the clinic to treat a wide range of cancer types, including NSCLC [23]. Significant differences in immunotherapy scoring mechanisms were revealed in two anti-PD1/L1-negative immunotherapy regimens, and the low GAPDH expression group had a higher antitumor therapeutic efficacy (Figure 6F,G). There was no discernible difference between the groups receiving anti-PD1/L1 immunotherapy with high or low GAPDH expression (Figure 6H,I).

### 3.6. GAPDH Expression Significantly Reduced during Ferroptosis

**Figure 7:**
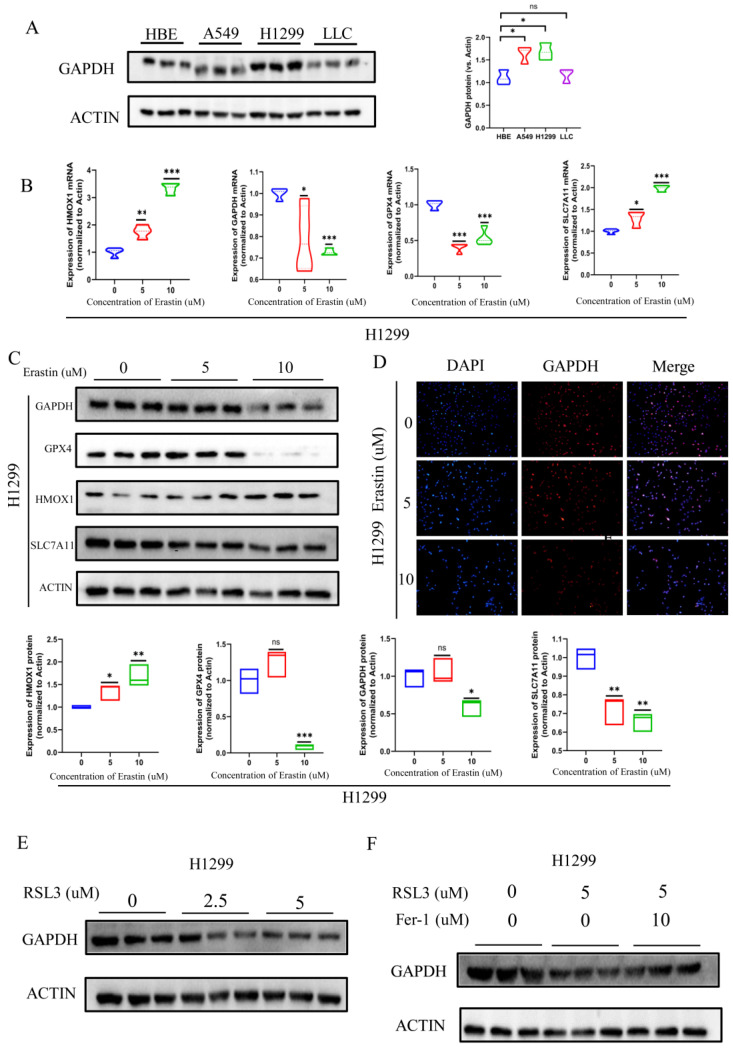
GAPDH levels declined during ferroptosis. (**A**) Western blotting showed GAPDH expression in HBE, H1299, A549, and LLC cells. (**B**,**C**) RT-qPCR quantitative analysis (**B**) and Western blot analysis (**C**) of GPX4, SLC7A11, HMOX1, and GAPDH expression in H1299 cells treated with erastin, 5 or 10 μM. (**D**) Immunofluorescence plot analysis of GAPDH expression in H1299 cells treated with erastin, 5 or 10 μM. (**E**) GAPDH expression was downregulated in H1299 cells with RSL3 (2.5 or 5 μM) treatment, verified by Western blotting. (**F**) Fer-1 (10 μM) reversed RSL3 (5 μM)-induced GAPDH reduction, verified by Western blotting. Statistical analysis was performed using Student’s *t*-test (ns, not significant, * *p* < 0.05, ** *p* < 0.01, *** *p* < 0.001).

GAPDH is a key enzyme in glucose metabolism, and its expression differs between tumor cells and normal cells (Figure 7A). Naturally, we wondered whether GAPDH expression changes during ferroptosis progression, as suggested by the bioinformatics analysis. Erastin, a cell-permeable compound, was discovered to be the first chemical to cause cancer cells to undergo ferroptosis in 2012 [24]. Since then, erastin has been utilized in numerous studies to induce ferroptosis. The toxicity of erastin in human lung adenocarcinoma cells (H1299 and A549) was further examined. Consistent with what was previously reported, erastin showed a dose-dependent relationship with toxic effects in tumor cells (Figure S4A). We detected the expression of crucial molecules in the ferroptosis pathway in both H1299 and A549 cells, including GPX4, HMOX1, SLC7A11, and especially GAPDH. Compared with the control group, cells from the erastin-treated group had significantly decreased levels of GPX4, SLC7A11, and GAPDH, with HMOX1 considerably increased, at both mRNA and protein levels (Figure 7B,C and Figure S4B,C). Following that, immunofluorescence findings revealed significantly reduced GAPDH protein levels in both H1299 and A549 cells (Figure 7D and Figure S4D). Additionally, RSL3 (a Gpx4 inhibitor) treatment of H1299 cells also resulted in lower GAPDH protein expression (Figure 7E). The anticipated elevation of GAPDH protein expression was caused by ferrostatin-1’s capacity to counteract RSL3-induced lipid peroxidation and cell death (Figure 7F). Similar to this, RSL3 treatment also reduced the expression of GAPDH in A549 cells (Figure S4E). To sum up, GAPDH expression changed as ferroptosis developed and eventually decreased. This suggests that GAPDH might be a brand-new indicator of ferroptosis in LUAD.

## 4. Discussion

A unique form of programmed cell death called ferroptosis is primarily brought on by iron-dependent lipid peroxidation [25]. The few ferroptosis-related studies in lung cancer largely concentrated on the possible function of ferroptosis biomarkers in the ferroptosis-inducing process of known inducers [26,27,28]. There is a lack of thorough and methodical investigation of ferroptosis markers in LUAD evolution and therapy.

In this investigation, we found 60 FRGs linked to patient survival by methodically analyzing the expression profiles and prognostic values of 638 ferroptosis-related genes obtained from the GeneCards and OMIM databases. Then, utilizing the method of the LASSO Cox regression model, a ferroptosis-related prognosis risk model with 12 genes was established. A subgroup evaluation of several clinicopathological features supported the stable prediction by the FRRM. According to the FRRM, 409 genes differentially expressed in high- and low-risk categories were identified in our study as potential prognostic ferroptosis-related genes. With the assistance of a PPI network, GAPDH was selected as a potential ferroptosis marker for further investigation. In 23 malignancies, GAPDH was considerably different, and all 23 tumors had significantly higher GAPDH levels than normal tissues. The Wilcoxon rank sum test revealed that GAPDH expression was considerably higher in LUAD tumor samples than in normal samples, and similar results were observed when normal and tumor tissues from the same patient were analyzed together. Survival curve analysis showed that the high GAPDH expression group had a worse survival rate. In a subsequent analysis of the association between GAPDH and clinical characteristics, we found that GAPDH expression was higher in male patients, and GAPDH was positively associated with tumor metastasis and progression.

Apart from clinical features, we wondered whether GAPDH expression was associated with the tumor immune microenvironment. Immune cell infiltration analysis revealed that the high GAPDH expression group was enriched in activated memory CD4 T cells, M0 macrophages, M1 macrophages, resting NK cells, and activated mast cells, while monocytes, resting dendritic cells, memory B cells, resting mast cells, and resting memory CD4 T cells were higher in the low GAPDH expression group. Additionally, the majority of immune checkpoint proteins, such as BTLA, TNFSF15, CD160, NRP1, TNFRSF14, ADORA2A, CD28, CD40LG, CD200R1, and CD48, were found to be negatively correlated with GAPDH expression; only a small number, such as CD70, CD274, LAG3, and CD276, were discovered to be positively correlated with high GAPDH expression. Furthermore, the number of tumor mutations is strongly linked to high GAPDH expression. Anti-PD-1 therapy did not reveal any appreciable variations between subgroups.

To kill or suppress tumor cells, mainstream tumor chemotherapies currently use apoptosis-inducing modalities [29]. The likelihood of treatment failure and posttreatment relapse has significantly increased as a result of tumor cells’ ability to exhibit intrinsic or acquired resistance to these apoptosis-dependent antitumor modalities [30,31]. By strengthening the immune systems and the anticancer immune response, cancer immunotherapy tries to combat tumor cells [32]. The immune response involves a cycle that includes the release and presentation of tumor antigens, the activation of lymphocytes, and the recognition and elimination of cancer cells [33]. However, the majority of currently available immune-based tumor treatments are ineffectual. Tumors’ low immunogenicity and immunosuppressive microenvironment are detrimental to both starting and enhancing the immune cycle [34]. Immunotherapies frequently induce severe toxic side effects because of highly activated T cells assaulting normal body tissues and generating a significant amount of proinflammatory cytokines [35]. Immune checkpoint inhibitors, for instance, have been shown to make patients more susceptible to pulmonary toxicity [36]. It is urgent to devise a workable plan for an immune therapeutic application that is safe and efficient in light of all of these adverse occurrences. Therefore, finding new non-apoptotic pathways that control cell death and enhance the immune microenvironment of malignancies is crucial from a clinical and practical standpoint [37].

In the context of a complicated triangular relationship between pathological cell death, inflammatory responses, and immunological responses, ferroptosis responses take place [38,39]. Due to the rapid proliferation and metabolic rate acceleration of tumor cells, tumor energy metabolism becomes a target for the disturbance of redox homeostasis and the induction of ferroptosis [40]. Deregulated cellular energetics is a hallmark of tumors [41]. Aerobic glycolysis is mostly used by solid tumors to fuel their rapid growth, known as the “Warburg effect” [42]. There is an intense metabolic competition between tumor and immune cells in TIME [43]. Higher expression and activity of glycolytic enzymes are associated with increased glucose uptake and accelerated glycolysis in tumor or immune cells, whereas glucose use via oxidative phosphorylation is decreased [18,44]. Tumors could exhaust glucose and accumulate lactic acid in TIME, create a nutrient-limited environment, and blunt T cells to finally limit the reinvigoration of antitumor immunity [45,46]. The formation and accumulation of dendritic cells, the cytotoxic activity of T cells, and the survival and inhibitory actions of the suppressive functions of regulatory T cells may all be hampered by the high lactate levels in TIME [46,47]. Inhibiting aerobic glycolysis in the tumor microenvironment by targeting GAPDH, a glycolytic pathway shared by both tumor and immune cells, normalizes trophic competition in TIME and reduces the cytotoxic side effects of immunotherapy [43]. This might be a desirable tactic for boosting antitumor immunity. Furthermore, tumors with higher glycolytic activity had weaker T-cell responses [43]. Three important glycolysis enzymes, HK II, platelet-type phosphofructokinase, and pyruvate kinase M2 (PKM2), were found to be significantly downregulated in tumor cells that were undergoing ferroptosis induced by erastin or RSL3 [48,49]. The possible mechanism is by switching the cellular metabolic pathway from glycolysis to oxidative phosphorylation (OXPHO), leading to a burst of intracellular reactive oxygen species (ROS) [40]. Conversely, it has been shown that glucose-6-phosphate dehydrogenase was increased in breast cancer cells that were undergoing ferroptosis induced by erastin, and this may be seen as a compensatory mechanism [50]. Glycolysis inhibition is coupled with greater sensitivity to conventional chemotherapy and inducers of ferroptosis [51]. This reprograms TIME and promotes the antitumor immune cycle. Macrophages successfully transition from the progenitor M2 to the antitumor phenotype M1 through ferroptosis, which also improves metabolic programming (from OXPHO to glycolysis) and activates several proinflammatory signals [52]. These insights directly demonstrate the close and direct link between glycolysis and ferroptosis.

We next examined the changes in GAPDH expression during the ferroptosis process in vitro experiments. We induced ferroptosis in H1299 and A549 cells by erastin and RSL3 and found that the expression of GAPDH decreased notably with the progression of ferroptosis. The reduction in GAPDH expression was prevented by the ferroptosis inhibitor ferrostatin-1. Additionally, GAPDH knockdown boosted Fe^2+^-induced ferroptosis within tumor cells [21]. These results imply that ferroptosis affects glycolysis levels in lung cancer cells and that GAPDH may serve as a prognostic sign for lung cancer patients as well as a marker of ferroptosis. However, this study still has a lot of limitations. The conclusions of these analyses are supported by publicly available datasets and our experiments. More clinical or independent cohorts need to be checked, and additional in vivo and in vitro experiments are required to confirm these results.

## 5. Conclusions

In summary, we constructed a ferroptosis-related risk model consisting of 12 key prognostic DEGs by methodically analyzing the expression patterns and prognostic significance of 60 ferroptosis-related genes in LUAD patients. Our prognostic model’s effectiveness was later examined by external validation cohorts, and it was found to be a reliable predictor of outcome for LUAD patients. After that, GAPDH was proved to be the key ferroptosis gene in a PPI network. In vitro experiments further supported the potential role of GAPDH as a ferroptosis marker in LUAD cell lines. However, additional experimental verification is still required to determine the precise function and mechanism of GAPDH in LUAD.

## Data Availability

The datasets generated and analyzed during this study are available in the TCGA database (https://portal.gdc.cancer.gov, accessed on 1 May 2022).

## Supplementary Materials

The following are available online at https://www.mdpi.com/article/10.3390/metabo13020142/s1, Figure S1: The overall design and processes of the study. Figure S2: Ferroptosis-related risk model establishment. (**A**) Mutation co-occurrence and selection analysis for 60 ferroptosis-related genes. Khaki indicates exclusion, whereas green indicates co-occurrence. (**B**) LASSO coefficient spectrum of 12 ferroptosis-related genes. (**C**) Cross-validation of a proportional hazards model’s adjustment parameter choice. (**D**) PCA based on ferroptosis-related risk scores in the TCGA-LUAD cohort. (**E**) PCA based on ferroptosis-related risk scores in the GEO-LUAD cohort. Patients at high risk are represented by the red group, while those at low risk are represented by the blue group. Figure S3: (**A**) GSVA enrichment heatmap for excellent and poor score groups. (**B**) A protein–protein interaction network showing the interaction and subnetworks of the differentially expressed genes. Figure S4: (**A**) Representative phase-contrast images of H1299 and A549 cells treated with erastin, 5 or 10 μM. Scale bar: 50 um. (**B,C**) RT-qPCR quantitative analysis (**B**), and Western blot analysis (**C**) of GPX4, SLC7A11, HMOX1, and GAPDH expression in A549 cells treated with erastin, 5 or 10 μM. (**D**) Immunofluorescence plots analysis showed GAPDH downregulation in A549 cells treated with erastin, 5 or 10 μM. (**E**) GAPDH expression was downregulated in A549 cells with RSL3 (2.5 or 5 μM) treatment, verified by Western blotting. Statistical analysis was performed using Student’s *t*-test. (ns, not significant; * *p* < 0.05, ** *p* < 0.01, *** *p* < 0.001). Table S1: The sequences of primers for real-time PCR assays. Table S2: 638 ferroptosis-related genes found in GeneCards and OMIM database.
