# Supplementary material for: GAPDH Is a Novel Ferroptosis-Related Marker and Correlates with Immune Microenvironment in Lung Adenocarcinoma
Source: Metabolites. 2023 Jan 17;13(2):142. doi: 10.3390/metabo13020142 (PMC9961514; doi:10.3390/metabo13020142)
Supplement: Supplementary file 1 [file metabolites-13-00142-s001.zip › metabolites-2061062-supplementary.pdf]

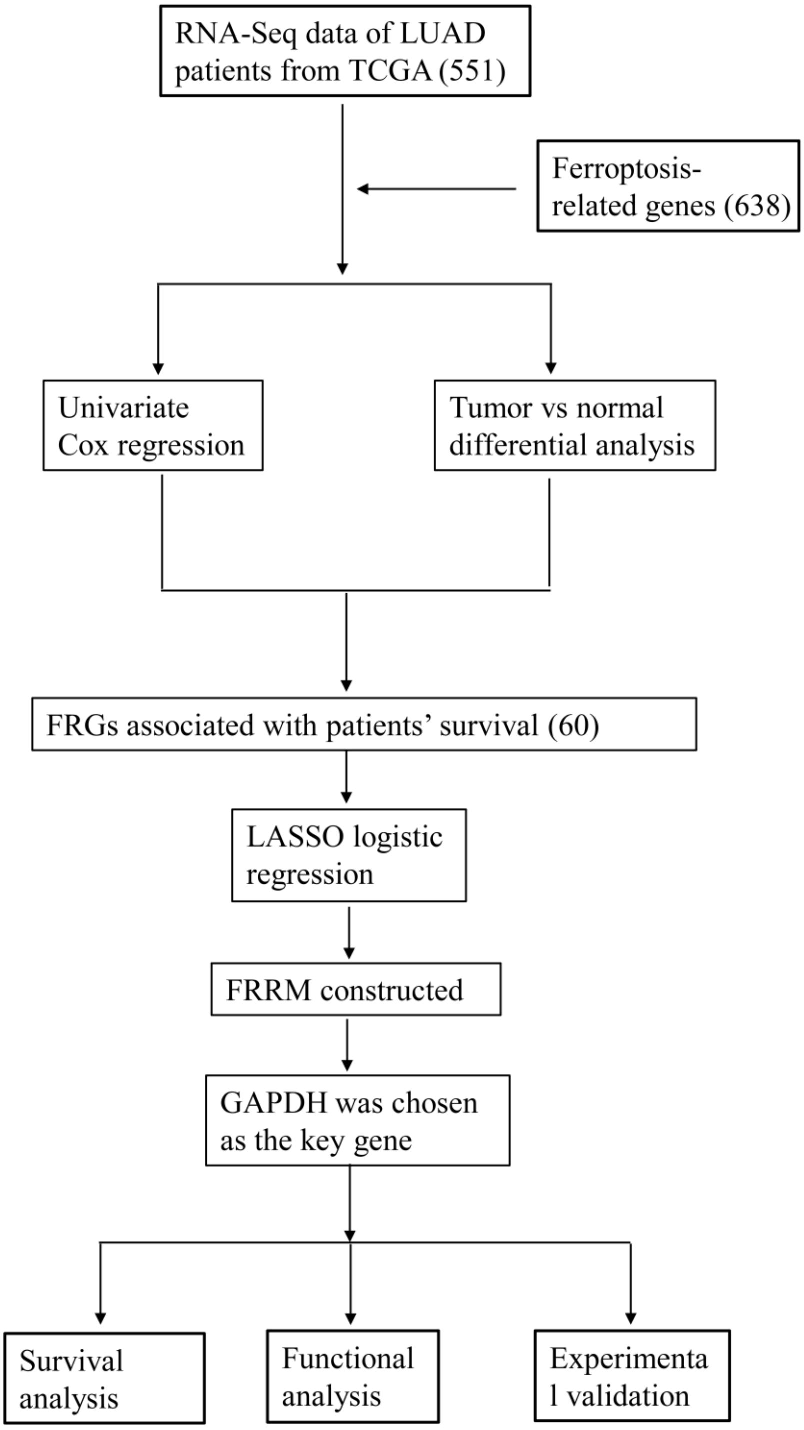

Figure S1. The overall design and processes of the study.

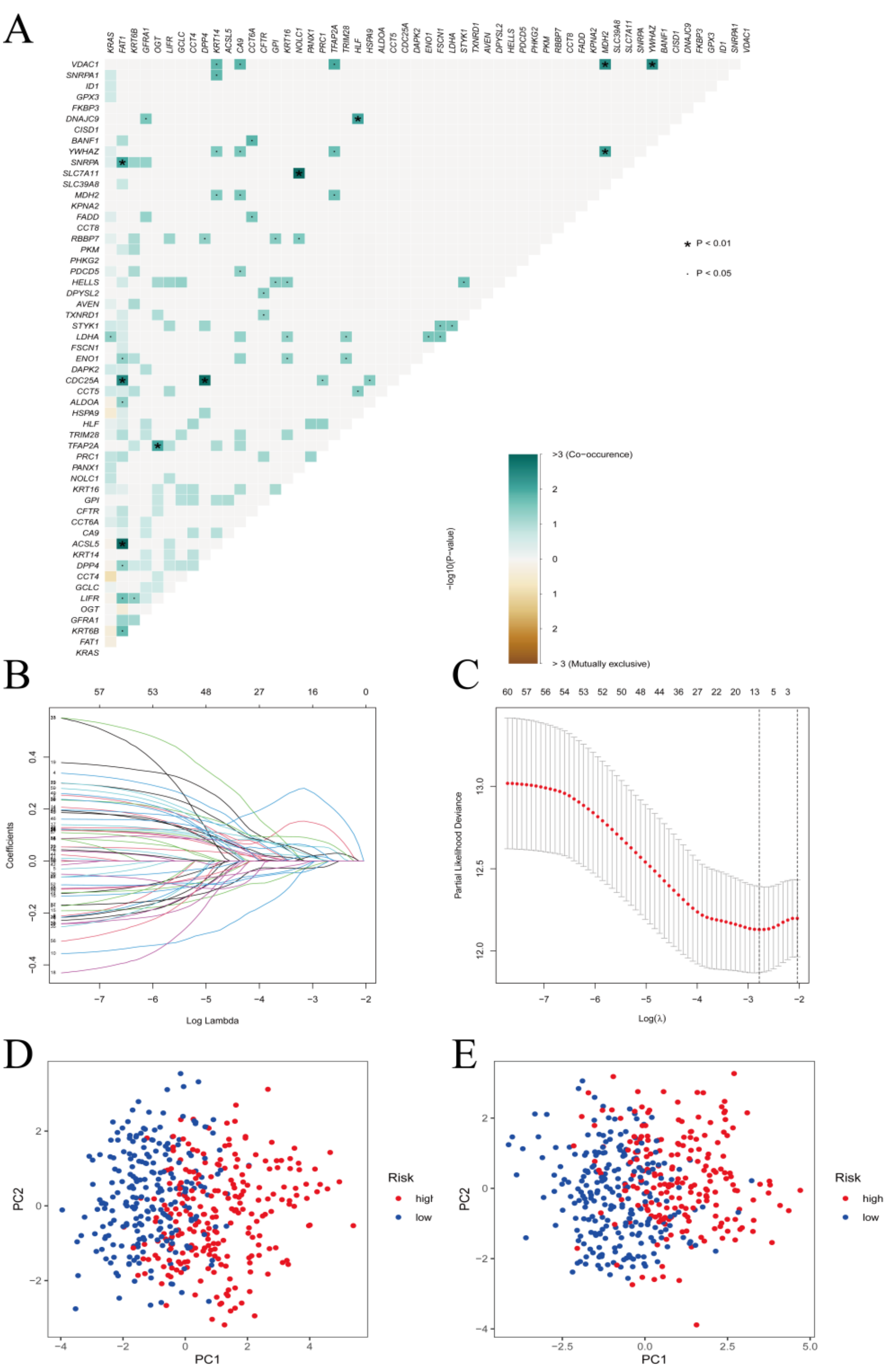

Figure S2. Ferroptosis−related risk model establishment. (**A**) Mutation co−occurrence and selection analysis for 60 ferroptosis−related genes. Khaki indicates exclusion, whereas green indicates co−occurrence. (**B**) LASSO coefficient spectrum of 12 ferroptosis−related genes. (**C**) Cross−validation of a proportional hazards model's adjustment parameter choice. (**D**) PCA based on ferroptosis−related risk scores in the TCGA−LUAD cohort. (**E**) PCA based on ferroptosis−related risk scores in the GEO−LUAD cohort. Patients at high risk are represented by the red group, while those at low risk are represented by the blue group.

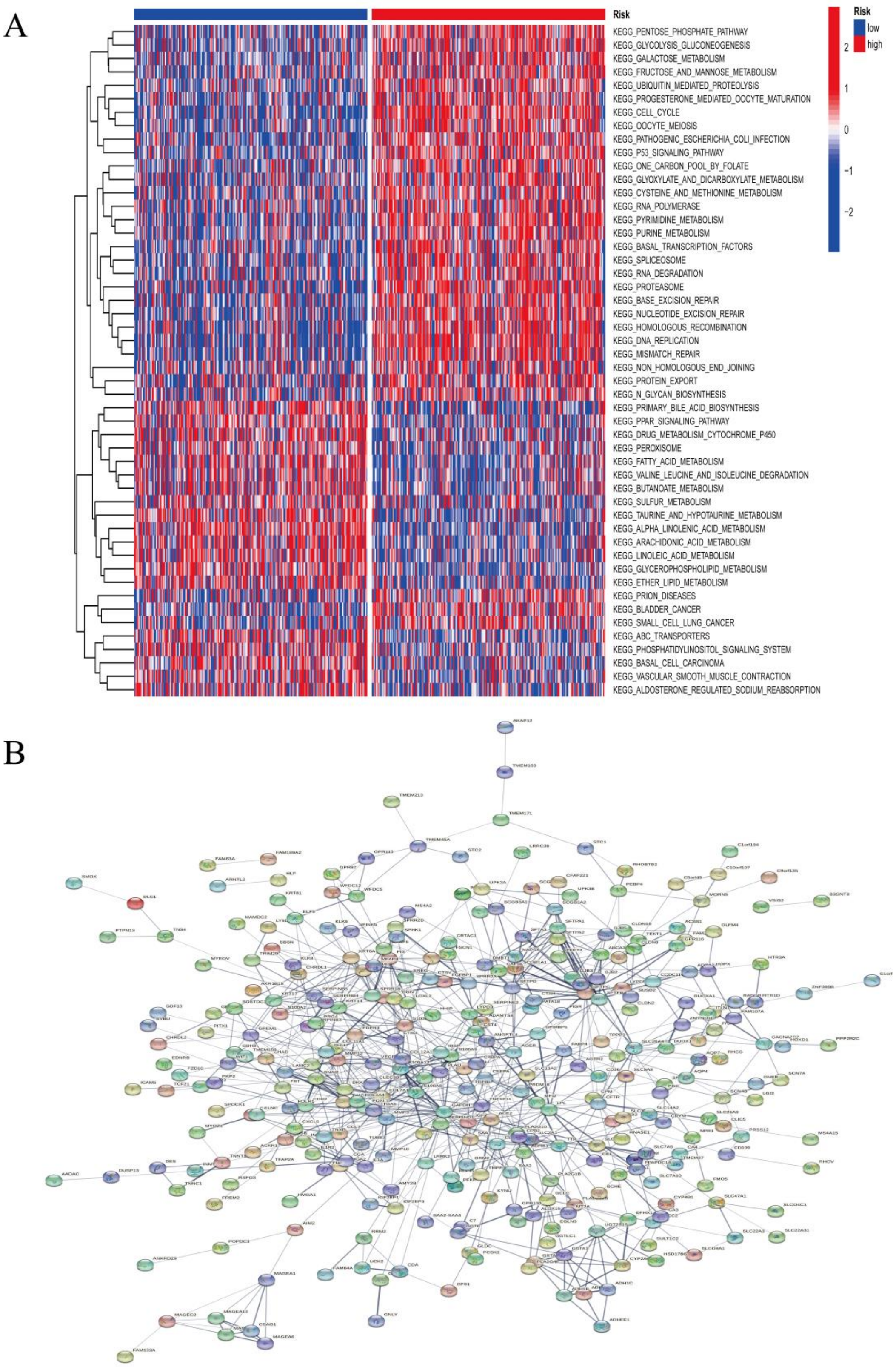

Figure S3. (**A**) GSVA enrichment heatmap for excellent and poor score groups. (**B**) A protein–protein interaction network showing the interaction and subnetworks of the differentially expressed genes.

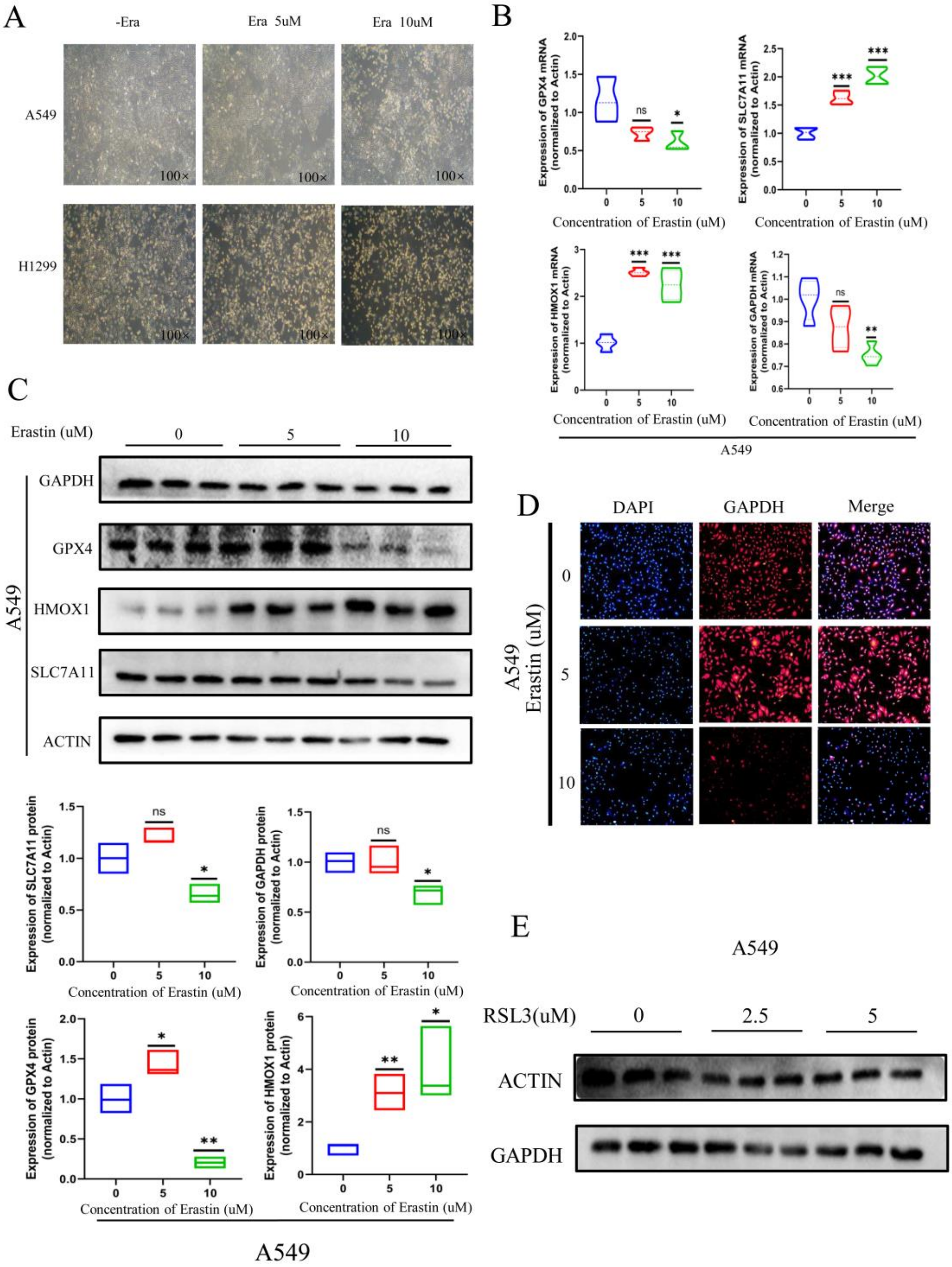

Figure S4. (**A**) Representative phase-contrast images of H1299 and A549 cells treated with erastin, 5 or 10 μM. Scale bar: 50 μM. (**B**,**C**) RT-qPCR quantitative analysis (**B**) and Western blot analysis (**C**) of GPX4, SLC7A11, HMOX1, and GAPDH expression in A549 cells treated with erastin, 5 or 10 μM. (**D**) Immunofluorescence plot analysis showed GAPDH downregulation in A549 cells treated with erastin, 5 or 10 μM. (**E**) GAPDH expression was downregulated in A549 cells with RSL3 (2.5 or 5 μM) treatment, verified by Western blotting. Statistical analysis was performed using Student's *t*-test. (*$p < 0.05$, **$p < 0.01$, ***$p < 0.001$).

Supplementary Table S1. The sequences of primers for real-time PCR assays.

Gene	Sequence
GAPDH-F	ATCATCCCTGCATCCACT
GAPDH-R	ATCCACGACGGACACATT
GPX4-F	CCTCCCCAGTACTGCAACAG
GPX4-R	GGCTGAGAATTCGTGCATGG
SLC7A11-F	TGGCGGTGACCTTCTCTGA
SLC7A11-R	ACAAAGATCGGGACTGCTAATGA
HMOX1-F	CCAGGCAGAGAATGCTGAGTTC
HMOX1-R	AAGACTGGGCTCTCCTTGTTGC
ACTIN-F	ACACCCGCCACCAGTTCGC
ACTIN-R	ATGGGGTACTTCAGGGTCAGGATA

Supplementary Table S2: 638 ferroptosis-related genes found in GeneCards and OMIM database.

AIFM2	CTH	ALOX5	EIF4H	TRIM28	CPS1	PDCD2
GPX4	CYBB	PTGS2	KRT6B	VAPA	EEF2	SLC25A37
SLC7A11	GCH1	KEAP1	PSME1	MAP4	GRN	ADHFE1
NFE2L2	SLC11A2	ABCB6	RPS2	RPL26	MIF	PDCD1L2
TP53	PHKG2	NEDD4	TXNL1	RPS13	SQSTM1	ACO1
ACSL4	PRNP	NEDD4L	CLTB	ATP5F1A	ABCA4	ISCA1
TFRC	GSS	FXN	MYL12A	CCT3	FHL2	DAPK1
HMOX1	HMGCR	ACADSB	PHB1	CCT4	HCFC1	CRAT
SLC3A2	STEAP3	LTF	PSMD8	DHX9	RPS6KB2	PDCD11
NCOA4	SLC1A5	PANX1	TPM4	HMGN1	COPB2	PDCD4
SLC40A1	SLC39A14	YME1L1	ANP32B	NOLC1	C1QBP	PIDD1
HSPB1	SLC39A8	ETV4	DECR2	PDIA4	MYO9B	MADD
FTH1	ACSL5	KDM4A	FKBP3	RPLP0	SF3B1	BAD
POR	NOX1	RHOT1	STOM	RPLP2	AHCYL1	FADD
ALOX15	MAP1LC3A	PLIN2	TPD52L2	RPS7	ASXL1	BLID
CBS	COQ2	CSRP2	DNAJC9	CCT6A	CBX5	CRADD
IREB2	GCLM	STOML2	TRIM26	CCT8	GPX2	ISCU
YAP1	PCBP2	DAZAP1	CARS1	HNRNPA3	HAT1	DAD1
FTMT	SLC38A1	GABPB1	CCDC124	HNRNPL	HUWE1	CIDEB
MAP1LC3B	ACSL6	H2AX	PYM1	PDIA6	KPNA2	ISCA2
SAT1	CHMP5	METTL14	MIR137	PFN2	MSRB3	AVEN
CP	SAT2	YTHDF2	MIR9-1	PLXNA4	OGT	DAPK2
CISD1	LPCAT3	SLIRP	CHAC1	RPL30	RPS10	PDCD7
AKR1C3	CHMP6	SCGB1D2	BEX1	SLC25A31	RUVBL2	CDIP1

AKR1C2	MAP1LC3C	TMED8	EPAS1	SNRPA	SPI1	OGFOD1
AKR1C1	GRIK1-AS2	MIR375	NFS1	SNRPD2	ARCN1	TRADD
BECN1	PSEN1	MIR106A	RB1	SSBP1	COPA	COASY
NOX4	SPARC	ASMTL-AS1	G3BP1	WTAP	DIAPH3	CIDEA
GCLC	AHCY	GABPB1-AS1	LINC00472	HNRNPM	EFTUD2	UQCRFS1
VDAC2	MYCN	NOTCH2	HIF1A	KSR2	KPNB1	C19orf12
VDAC3	FOXC1	TYRO3	HILPDA	MUC16	POLR2B	PDCD5
PARK7	STYK1	CA9	G0S2	MYL6B	POLR2E	PDCD2L
ATG5	PIR	FZD7	BCAT2	NACA	RBBP4	DEDD2
ATG7	RBMS1	SIRT6	HSPA5	NGB	USP11	PANK2
ATF4	SESN2	SQLE	GDPD5	RPL34	ALOX15B	DATF1
PRKAA1	PVT1	ABCC5	MIR874	RPS4X	ANO6	BID
ACSL1	MIR670	BSG	TIGAR	RUFY1	CISD2	HSC20
PCBP1	VDAC1	TTBK2	MTOR	SNRPD1	ID1	TMPRSS6
LCN2	IDH2	ITGB8	PRC1	SNRPF	PRPF8	
TF	NFKB1	COPZ1	HADHA	USP44	RB1CC1	
MIR214	DLD	HLF	HSPA9	YBX1	RBBP7	
SIRT3	IL6	MEG8	RUVBL1	ALYREF	RPS12	
HMGB1	JUN	MIR129-1	CBX3	ATP5F1B	RPS27A	
ELAVL1	KAT5	MIR497	NAP1L1	CNTNAP4	ALDH1L2	
WWTR1	DKK1	LINC01833	MT1G	FAM120A	COPB1	
ATF3	LIFR	MIR4443	H19	RPL38	DNAJA1	
OTUB1	HNRNPA1	MIR761	MIR106B	SNRPC	DNAJA2	
KRAS	SOX2	EGFR	TGFBR1	YTHDF3	FAT1	
PROM2	STUB1	PDGFRB	UCHL1	ERH	ID3	
LINC00336	MDM4	FABP4	GSTP1	PCBP3	MAGED2	
HELLS	GPX3	DNAJB6	SNCA	TBC1D5	PCMT1	
LAMP2	ARNTL	DPEP1	VIM	WBP11	RBM10	
SCD	GSTZ1	GALNT14	FASN	ACTBL2	RCN2	
GOT1	HCAR1	CDK14	MYH9	IGKC	RPS23	
DECR1	YTHDC2	CHMP1A	YWHAG	RPL36A	TNPO1	
ECH1	RETREG1	KLF2	ALDOA	RUFY3	ALDH1L1	
DHODH	USP35	QSOX1	ANXA1	SNU13	COPE	
STK11	MIR302A	MIR132	GPI	TEX264	GUCY1A1	
PLA2G6	MIR424	MIR1231	PGK1	ABRAXAS2	RPL24	
MDM2	DDR2	MIR3938	TUBA1A	KBTBD3	RPS15	
RELA	LONP1	ITGA6	ACVR1B	RTL1	UBE2O	
FTL	PDK4	PRKAA2	ENO2	KIAA1614	ASXL2	
TXNRD1	CD82	CD44	GNB3	H2AC21	COPG1	
FDFT1	ASAH2	FANCD2	LDHB	SRSF8	FOXK2	

ACSL3	MGST1	ALOX12	MYH10	IGHG4	NAP1L4
NF2	SMG9	NQO1	RPL5	TUBA4B	TXNIP
XRCC6	HDDC3	LDHA	YWHAB	IGKV1-5	COPG2
ATM	CISD3	MAPK1	YWHAZ	IGLV3-19	GOLGA4
TNF	EP300	MYC	DLST	NET1	KLHDC2
FBXW7	CREB1	PCNA	HP	MIR494	RNF113A
SKP2	ERCC6	ACTB	HSP90B1	LPIN1	HCFC2
ATG16L1	TFAP2A	ALB	PRDX2	NDUFS7	LANCL2
SLC39A7	KDM5C	EGLN1	UBA1	PDCD1	NAP1L5
ZFP36	PEBP1	ANXA2	CCT5	SIAH2	SNRPA1
H2AC1	UBE2D3	HSPA8	CFL1	LPIN2	STING1
BAP1	DDB1	KRT18	CKB	CIAO1	ANKHD1
MEF2C	KDM3B	MDH2	DPYSL2	LPIN3	ELOC
ADCY10	CCL5	TPM1	EIF4A1	CIAO2A	FOXK1
ERBB2	CUL4A	CTSZ	RPL10	OTUD1	GCN1
CTSB	CUL4B	ENO1	RPSA	TGFB2	FOXI3
YWHAE	RBX1	KRT14	SMC3	PKN2	FOXI2
CDC25A	SETDB1	MAP3K5	TUBB4A	GFRA1	MIR7-1
PRDX6	ABCB10	TPM3	VAPB	UBR2	SDHB
PKM	CYB5R1	ANXA5	ACTC1	MIR194-1	SLC39A1
SP1	PDSS2	FH	EWSR1	MIR1228	DAP3
RPL7	CYGB	GNB1	PDIA3	CDKN2A	DEDD
RPS3A	DCAF8	KRT19	PROK2	AURKA	IBA57
RACK1	H1-4	LGALS3	RPA2	RIPK1	EDARADD
MIR30E	H1-5	PRKCSH	RPL15	CASP8	NFU1
IDH1	H1-2	ANXA4	XRCC5	SF3B2	EVA1A
PTEN	PAQR3	ATP6V1E1	BANF1	MIR4715	CIDEC
GJA1	H1-1	KRT16	CNBP	CHEK2	PDCD6IP
GSK3B	H1-3	SOCS1	DUT	CFTR	SCN5A
CAV1	WDR76	ANP32A	FSCN1	GNAS	PDCD10
PRDX1	H1-10	EEF1A1	HNRNPD	TGFB1	DAP
TNFAIP3	MIR326	PSMA1	ITIH3	CAD	DELE1
PRKCB	MIR324	RPL7A	KHDRBS1	FOXO1	HFE
SREBF1	LASTR	RPS3	LRPPRC	MUC1	DAXX
NEAT1	BACH1	RPS6	PTBP1	NPM1	TSPYL1
DPP4	STAT3	SNAP29	RPL13A	VCP	REPS1
